# Does the Pre-Ovulatory Pig Oviduct Rule Sperm Capacitation In Vivo Mediating Transcriptomics of Catsper Channels?

**DOI:** 10.3390/ijms21051840

**Published:** 2020-03-07

**Authors:** Cristina A. Martinez, Manuel Alvarez-Rodriguez, Dominic Wright, Heriberto Rodriguez-Martinez

**Affiliations:** 1 Department of Biomedical and Clinical Sciences (BKV), BKH/Obstetrics and Gynecology, Faculty of Medicine and Health Sciences, Linköping University, SE-58185 Linköping, Sweden; manuel.alvarez-rodriguez@liu.se (M.A.-R.); heriberto.rodriguez-martinez@liu.se (H.R.-M.); 2 Department of Physics, Chemistry and Biology, Faculty of Science and Engineering; Linköping University, SE-58183 Linköping, Sweden; dominic.wright@liu.se

**Keywords:** capacitation, oviduct, sperm oviduct reservoir

## Abstract

Spermatozoa need to conduct a series of biochemical changes termed capacitation in order to fertilize. In vivo, capacitation is sequentially achieved during sperm transport and interaction with the female genital tract, by mechanisms yet undisclosed in detail. However, when boar spermatozoa are stored in the tubal reservoir pre-ovulation, most appear to be in a non-capacitated state. This study aimed at deciphering the transcriptomics of capacitation-related genes in the pig pre-ovulatory oviduct, following the entry of semen or of sperm-free seminal plasma (SP). Ex-vivo samples of the utero-tubal junction (UTJ) and isthmus were examined with a microarray chip (GeneChip^®^ Porcine Gene 1.0 ST Array, Thermo Fisher Scientific) followed by bioinformatics for enriched analysis of functional categories (GO terms) and restrictive statistics. The results confirmed that entry of semen or of relative amounts of sperm-free SP modifies gene expression of these segments, pre-ovulation. It further shows that enriched genes are differentially associated with pathways relating to sperm motility, acrosome reaction, single fertilization, and the regulation of signal transduction GO terms. In particular, the pre-ovulation oviduct stimulates the Catsper channels for sperm Ca^2+^ influx, with *AKAPs, CATSPERs,* and *CABYR* genes being positive regulators while *PKIs* and *CRISP1* genes appear to be inhibitors of the process. We postulate that the stimulation of *PKIs* and *CRISP1* genes in the pre-ovulation sperm reservoir/adjacent isthmus, mediated by SP, act to prevent premature massive capacitation prior to ovulation.

## 1. Introduction

The complex nature of maternal-gamete communication has gained increased interest over the past decades. It is known that the signals provided by the gametes and interactions with the female genital tract are capable of rapidly influencing the local environment, stimulating a broad range of events towards successful fertilization and subsequent pregnancy [1,2]. These signals are critical in influencing a wide number of gamete functions such as final gamete maturation, gamete transport and survival, and are initiated by the gametes themselves immediately after ovulation or through mating/artificial insemination [3]. These events are all dependent upon the relay of the appropriate molecular signals between the maternal environment and the gametes. On the male side, after spermatozoa are deposited in the female genital tract, they have to overcome multiple physical barriers and complex interactions until they reach the site of fertilization at the mid-ampulla [4]. The fact that semen deposition elicits molecular alterations in the female genital tract has been previously described [5,6,7,8]. It appears to evoke an inflammatory response towards the acquisition of the so-called maternal state of immune tolerance [9]. A recent study has demonstrated the activation of a local immune response in the endometrium and the oviduct as rapidly as 24 h after semen or even seminal plasma (SP) has been deposited in the porcine female genital tract [10].

Understanding the mechanisms regulating uterine/oviduct-gamete interactions prior to fertilization has attracted particular interest in recent years because of its potential relationship with infertility, or even its relation to the subsequent embryo development [11,12]. It is known that inseminated spermatozoa must undergo functional modifications that render them competent to fertilize the oocyte, a process called “sperm capacitation” [13] discovered more than 60 years ago [14] but its mechanisms are still not known in detail. It is generally accepted that the functional changes achieved during sperm capacitation occur during two events. Firstly, spermatozoa display a hyperactivation of their motility, enabling them to detach from the isthmic reservoir and become free in the lumen, to be further transported to the site of fertilization. Secondly, when spermatozoa reach the oocyte, interact with the zona pellucida (ZP), and subsequently undergo the acrosome reaction [15]. Capacitation involves complex modification in the molecular landscape of the sperm plasma membrane [16,17]. These processes have been mimicked in vitro during assisted reproductive procedures by sperm incubation in well-defined culture media, resulting in adequate rates of sperm penetration through the ZP and fusion with the oolemma, though this does not always result in the formation of a viable zygote [18,19,20]. Moreover, boar spermatozoa in vitro display unpredictable acrosome disruption as well as inadequate sperm motility patterns, both contributing significantly to variable outcomes of in-vitro systems (IVF) [21]. To date, multiple methods have been carried out to achieve in vitro sperm capacitation, such as stimulation of the sperm cell’s kinematics by the addition of bicarbonate to the culture medium. Bicarbonate is a major secretory component of the oviduct during capacitation and plays a pivotal role in facilitating the influx of Ca^2+^ through the sperm membrane [22], and it is widely reported as *conditio sine qua non* for sperm hyperactivation in vivo [23]. Moreover, seminal plasma (SP), which contains inhibitors of capacitation preventing sperm premature capacitation [24], is eliminated during in vitro fertilization [25].

In vitro selection of spermatozoa to improve IVF is unlikely to correspond to events occurring in vivo [19]. Under in vivo conditions, progressively motile spermatozoa are selected during their journey through the genital tract [26,27] and most SP is removed from the sperm surface by interaction with the female (washing with intraluminal fluid, etc.) before arrival to the site of fertilization. Oviduct lining cells are further able to either promote or prevent capacitation in a time-specific manner to ensure the availability of sufficient numbers of functional spermatozoa once the ovulated oocytes reach the ampulla [28,29]. However, how the female tract participates in the capacitation process remains to be fully elucidated, particularly during the pre-ovulatory period, where capacitation is apparently prevented [13,30,31].

Overall, increased knowledge of the gene pattern and molecular processes regulating the activity of the oviduct that contribute to successful fertilization could lead to a significant advance in reproductive assisted technologies. Therefore, the objective of this study was to decipher the transcriptomics of capacitation-related genes in the pig pre-ovulatory oviduct, mediated by the entry of semen or of solely sperm-free seminal plasma in an in vivo experimental layout.

## 2. Results

### 2.1. Gene Expression is Altered in the Pre-Ovulatory Oviduct after Semen or Sperm-Free SP Exposure

**Figure 1:**
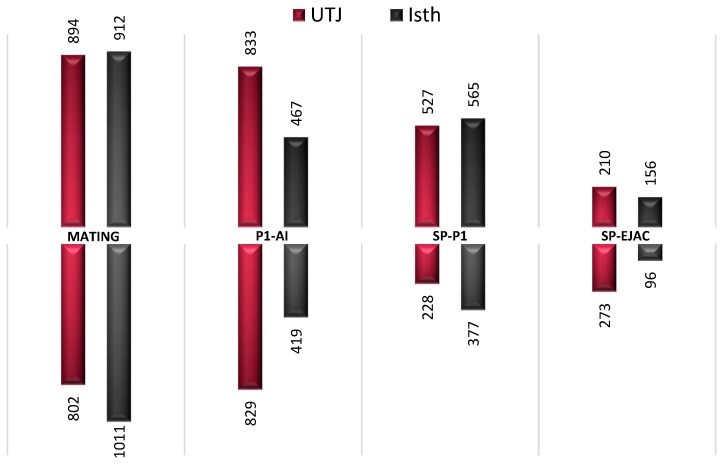
Differential expression (up- and down-regulation) of annotated genes. Treatment induced the differential expression of genes in all segments of the oviduct analyzed (Utero-tubal junction; UTJ, Isthmus; Isth). Mating: sow mated with a boar; P1-AI: sow artificially inseminated with the the sperm-peak ejaculate portion (P1) extended to 50 mL with BTS; SP-Ejac: sow cervically infused with sperm-free seminal plasma of the whole ejaculate (50 mL); SP-P1: sow cervically infused with sperm-free seminal plasma from pooled sperm-peak portion P1 (50 mL). All treatments were compared to Control (AI with 50 mL of BTS). The numbers represent the number of differentially expressed genes (*p-*value < 0.05).

The Affymetrix Porcine GeneChip^®^ (Thermo Fisher Scientific, Gothenburg, Sweden) was used for the transcriptomic analyses 24 h after semen (mating or P1-AI) or sperm-free (SP-P1 or SP-Ejac) exposure of the oviduct. Setting the fold change to 1/−1 and *p*-value to <0.05, the data analysis revealed a total of 1696 genes were differentially expressed (DEG) in the Utero-Tubal Junction (UTJ) and 1923 genes were differentially expressed in the isthmus (Isth) after mating (M), and 1662 (UTJ) and 886 (Isth) were DEG after artificial insemination (P1-AI), compared to controls (non-inseminated) sows. Exposure to sperm-free SP modified the expression of fewer genes; 755 (UTJ) and 942 (Isth) (SP-P1), and 483 (UTJ) and 252 (Isth) (SP-Ejac). Figure 1 shows the numbers of oviduct genes up- or down-regulated among groups. Overall, mating seemed to promote the highest differences in gene expression followed by P1-AI, SP-P1, and SP-Ejac.

### 2.2. Commonly Altered Genes between Pre-Ovulatory Oviduct Segments after Statistical Restrictive Analysis within Each Experimental Group

**Figure 2:**
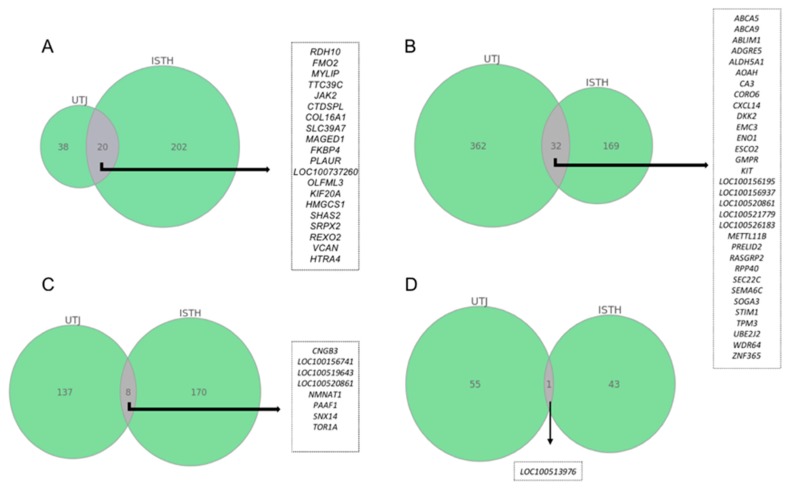
Venn diagram showing common altered genes among tissues within each treatment. Analysis of commonly altered transcripts among oviduct segments (UTJ: Utero-tubal junction; Isth: Isthmus;) after mating ((**A**); FDR < 0.05); artificial insemination from the sperm-peak ejaculate portion: P1-AI ((**B**); *p* < 0.01); exposure to seminal plasma from the sperm-peak ejaculate portion: SP-P1 ((**C**); *p* < 0.01); exposure to seminal plasma from the entire ejaculate: SP-Ejac ((**D**); *p* < 0.01). The numbers of genes altered in common are indicated at the intersections of the circles in the Venn diagram.

A more conservative statistical analysis was used to study the most robust data within each experimental group. The transcriptomic changes found were then plotted in a Venn diagram to visualize co-expressed genes shared among segments (Figure 2). Mating was the only group to show significantly altered transcripts using an False Discovery rate (FDR) < 0.05 analysis (**A**), sharing eight altered genes between the oviductal segments. P1-AI and sperm-free groups were analyzed according to an adjusted *p-*value of <0.01. The largest number of genes that altered their expression, common to both oviductal segments were registered in the P1-AI treatment (**B**), followed by the SP-P1 (**C**). Exposure to SP from the entire ejaculate (SP-Ejac group) did not yield changes of expression in genes common to either mucosal segment (**D**).

### 2.3. Analysis of Functional Categories: Enriched Tubal Genes During Pre-Ovulation are Differentially Associated with Sperm Motility, Acrosome Reaction, Single Fertilization, and Regulation of Signal Transduction

**Figure 3:**
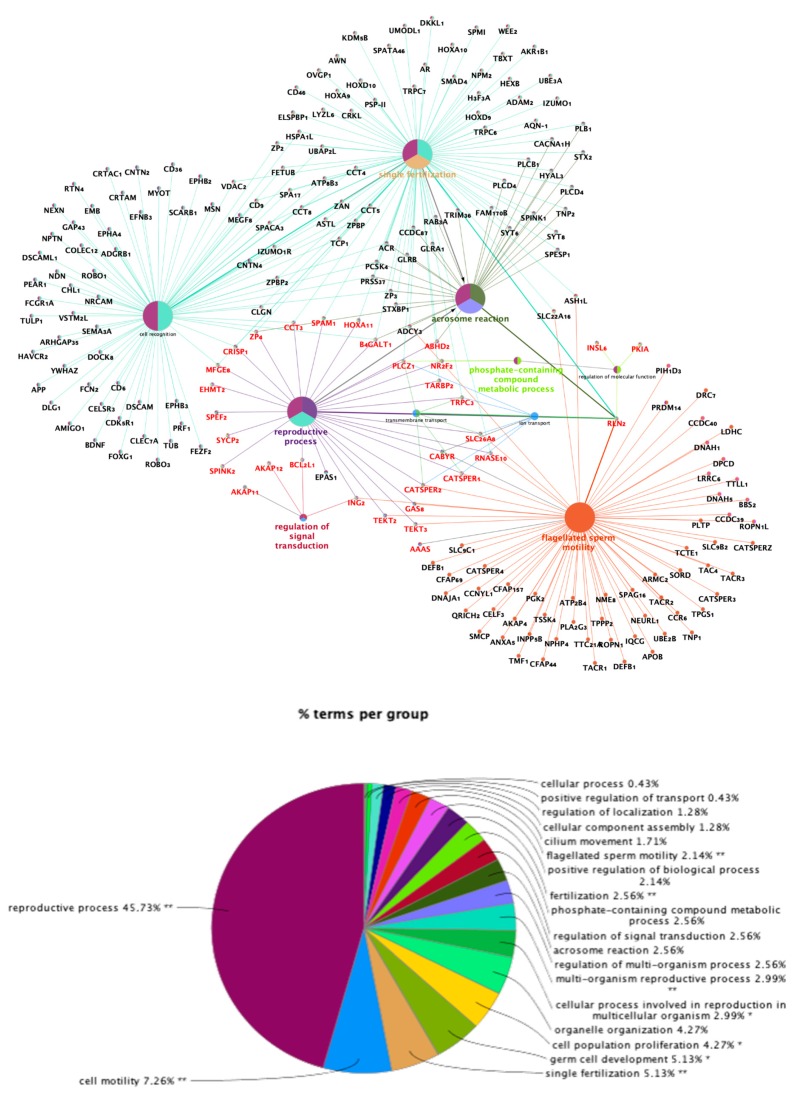
Schematic representation of functionally grouped terms. This network was created using the Cytoscape v3.0.0 application and the ClueGO+CluePedia (version 2.2.5) plug-in. Terms and their associated genes share the color. Genes marked in red are overrepresented in our study. The size of the nodes indicates the degree of significance, where the biggest nodes correspond to highest significance. The parameters included: biological process database (BP; date: 28.03.2019); GO tree levels, 2–6 (first level = 0); minimum number of genes, 3; minimum percentage of genes, 4; GO term fusion; GO term connection restriction (kappa score), 0.4; GO term grouping, initial group size of 2 and 50% for group merge; number of genes included in term <100. The resulting network was modified; that is, some redundant and noninformative terms were deleted and the network manually rearranged.

From the total gene set, we identified a subset of significantly differentially expressed transcripts involved in different biological processes that had potential roles in sperm capacitation. The transcripts of interest are shown in Table 1 (Mating: 20-UTJ; 17-Isth and P1-AI: 12-UTJ; 7-Isth groups) and Table 2 (SP-P1: 6-UTJ; 6-Isth and SP-Ejac: 3-UTJ; 1-Isth groups). The Database for Annotation, Visualization, and Integrated Discovery (DAVID 6.7) was used to annotate biological terms and processes preferentially represented in our study. The representation of those functional genes that were enriched for traits closely associated with sperm motility, acrosome reaction, single fertilization, and regulation of signal transduction, is shown in Figure 3.

### 2.4. Antagonistic Influences of Sperm- or Seminal Plasma on Pre-Ovulatory Oviductal Gene Expression for Sperm Capacitation Genes

**Figure 4:**
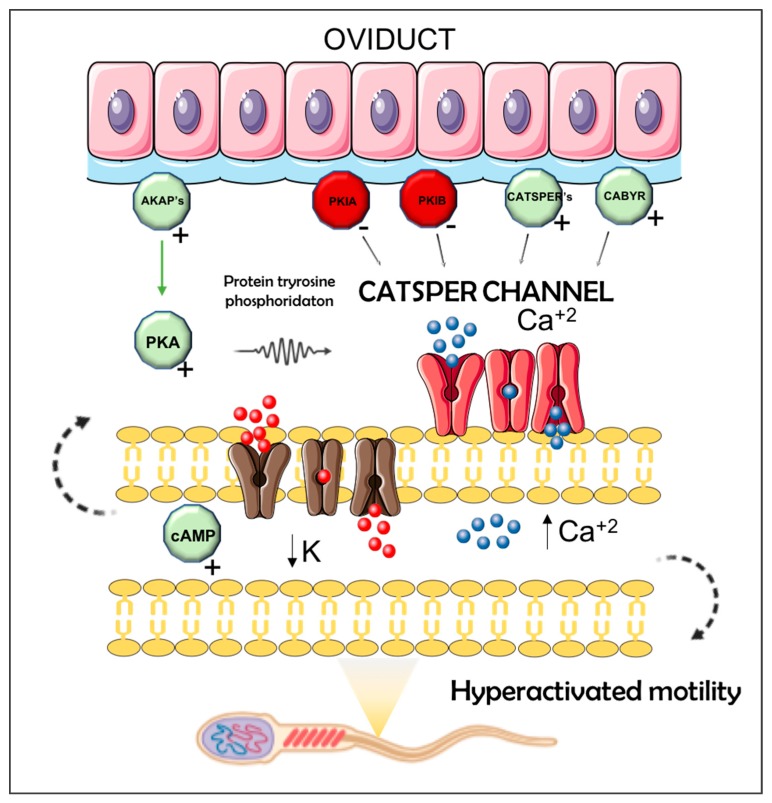
Model for sperm CatSper channel regulation in the oviduct. Possible signal transduction mechanisms of porcine sperm Ca2+ influx through the CatSper channels. The oviductal molecular signaling stimulate the receptors for spermatozoa Ca2+ influx in a positive or a negative manner depending on sperm or seminal plasma exposure. The different factors that are possibly affecting Ca^2+^ entry are depicted in the diagram, whereas AKAPs, CATSPERs, and CABYR genes are positive regulator of the Catsper channel, while PKIs and CRISP1 genes are inhibitors of the Catsper channels.

We found a strong enrichment of genes in the pre-ovulatory pig oviduct associated with sperm capacitation through Catsper channels regulation, where semen (M or P1-AI), via the contents of spermatozoa, was inducing pro-capacitation responses while sperm-free SP (SP-P1 and SP-Ejac) appeared to be promoting anti-capacitation responses. Among the transcripts potentially involved in Catsper signaling, we found an up-regulation of *CATSPER2* (UTJ) and *CATSPERγ* (UTJ and Isth) in the mating group and an up-regulation of *CATSPERγ* (Isth) in the P1-AI group (Table 1). On the other hand, we found *CATSPER1* (UTJ) downregulated in sperm-free SP group (SP-Ejac) (Table 2). Also, several members of the *AKAP* (A-Kinase anchoring proteins) family and the *CABYR*, *CIRP1*, *ABDH2*, *CRISP1*, and *RNASE10* genes, all with potential roles in regulating Catsper channels were altered in our study (Table 1 and Table 2). A model for the possible mechanisms of action of semen or SP, either from the entire ejaculate or its vanguard sperm-peak portion, in regulating oviduct signaling towards the activation or inactivation of these channels is presented in Figure 4. The model speculates that the pre-ovulation oviduct stimulates the receptors for sperm Ca^2+^ influx in a positive or a negative manner depending on whether it is exposed to spermatozoa or relative amounts of SP. In this case, *AKAPs*, *CATSPERs*, and *CABYR* genes are positive regulators while *PKIs* and *CRISP1* genes are inhibitors of the Catsper channels.

## 3. Discussion

To achieve fertilization competence, a spermatozoon must undergo an as yet incompletely understood series of complex morphological and molecular maturational mechanisms, termed capacitation. This involves, among other processes, protein tyrosine phosphorylation and increments in intracellular calcium. The expression of hyperactivated motility and an ability to undergo the acrosome reaction serve, even when appearing independently from each other, as physiological end points to assess successful capacitation. In the present study, we report, to the best of our knowledge for the first time, how exposure to semen (Mating or AI) or to sperm-free SP (SP-P1 or SP-Ejac) influences the pattern of transcription of genes related to sperm capacitation in each of three segments of the pre-ovulatory pig oviduct (UTJ and Isthmus) 24 h after in vivo experimental exposure. In spermatozoa, capacitation, hyperactivation of motility, and acrosome reaction when exposed to ZP-proteins, are all mediated by increases in intracellular Ca^2+^, a basic key signaling element [23]. The change from basal, activated motility in ejaculated spermatozoa to a display of hyperactivated motility is triggered by a rapid rise of intracellular concentration of Ca^2+^ through the Catsper channel (sperm-specific cation channel) [32]. This multi-protein complex contains four pore-forming subunits (Catsper1–4) and five accessory subunits called β, δ, ε, γ, and ζ encoded by at least nine genes in mammals [33,34] and it is known to play a pivotal role in Ca^2+^ signaling by allowing the transport of Ca^2+^ into the cell [35], thereby influencing sperm motility in all species explored thus far, including the pig [36].

Genetic evidence shows that the function of Catsper channel proteins is indispensable for male fertility. In mice, knocking-out of any of the four Catsper genes results in male infertility, with phenotypic impairment including sperm motility, loss of hyperactivated motility and failure to penetrate the ZP [33]. In human, genetic lesions and altered expression profiles of Catsper genes have been clinically linked to astheno-teratospermia and male infertility [37]. Catsper proteins have been widely described in the principal piece of the sperm tail [36,38,39,40], *CATSPERγ* and *CATSPERδ* subunits have been already identified in oviduct extracellular vesicles [41]. In the present study, we observed a higher expression of the *CATSPER2* gene in the UTJ, the main sperm reservoir, after mating when compared to controls. Additionally, we found the Catsper channel auxiliary subunit gamma (*CATSPERγ*), necessary for Catsper channel function, to be upregulated in both oviduct segments explored (UTJ and ISTH) after mating but only in ISTH after AI with the vanguard sperm-peak fraction (P1). To this moment, the mechanisms by which oviduct cells regulates Catsper channels are poorly understood. Recent work suggests that Catsper channels, and therefore, the main entry of Ca^2+^ into the spermatozoa, are activated by protein kinase A (PKA) via cAMP-dependent tyrosine phosphorylation [42,43,44]. In this sense, it has been reported that association of PKA to cellular structures is triggered by A-Kinase anchoring proteins (AKAPs), which are a group of structurally diverse proteins binding to the regulatory subunit of protein kinase A (PKA) to transfer PKAs to subcellular locations [45]. Disruption of the PKA–AKAP interaction seems to affect sperm attributes associated to fertilizing capacity, suggesting that AKAPs may be part of a signal transduction pathway also likely to be involved in sperm hyperactivation [46,47]. In the present study, we observed an alteration of many AKAP-encoding genes among oviductal compartments and treatments. For instance, expression of *AKAP11*, *AKAP12,* and *AKAP13* were significantly higher in most tubal compartments in response to semen entry (mating or AI) compared to the saline control (mating: UTJ-*AKAP11*, *AKAP13*; Isth-*AKAP11*, *AKAP12*; AI: UTJ-*AKAP11*, *AKAP13*). Moreover, PKA inhibitors were found down-regulated in the UTJ and Isth (*PKIA*) after mating, and down-regulated in the UTJ (*PKIB*) after AI of the P1 ejaculate fraction. Altogether, these findings indicate that PKAs are actively regulated 24h after semen exposure, when spermatozoa are mostly stored in the oviductal reservoir [48] and where sperm hyperactivation or acrosome burst are not taking place. Sperm are prevented from hyperactivation by several mechanisms [29], with capacitation not completed in most spermatozoa [30]. Numerous different AKAPs have been identified from various tissues and species [49]. Most of these A-Kinase anchoring proteins have been exclusively identified in testicular germ cells and mature spermatozoa [45,50]. Here we report for the first time evidence of their presence in oviductal samples of domesticated pigs. Moreover, among proteins phosphorylated via PKA during capacitation, we found an upregulation of *CABYR* (calcium-binding tyrosine phosphorylation-regulated gene) in the UTJ and Isth segments of the pig oviduct in response to semen exposure (mating). The protein encoded by this gene exhibits calcium-binding when phosphorylated during capacitation. It has been demonstrated that *CABYR* increases during in vitro capacitation, and calcium binding to these acidic forms is abolished by alkaline phosphatase dephosphorylation [51]. Although it has been widely described in the principal piece of the sperm flagellum in several species [52,53,54], *CABYR* is expressed in human oviduct cells [55]. However, we could not find any evidence that this gene has been previously reported in the porcine oviduct. Our results suggest that *CABYR* expression increases in the oviduct in response to sperm entry, possibly later contributing to changes in sperm flagellar movement and the display of hyperactivation. In the context of Catsper channels regulation, progesterone activates Catsper channels in human testicular and epididymal spermatozoa by binding to the sperm-membrane receptor; α/β hydrolase domain-containing protein 2 (*ABHD2*) which acts removing Catsper inhibitors to allow the channel to open [56,57,58,59,60]. However, it has been described in mouse spermatozoa that Catsper channels are activated by an increase in intracellular pH [61,62], and further they do not react to progesterone in vitro [56]; a situation similar to what our group recently reported for pig spermatozoa [36]. In the mouse, *ABHD2* does not seem essential for male fertility [56], an assumption which would be backed up by our results, considering that *ABDH2* was downregulated in the pig UTJ and Isth after mating compared to the controls. These overall findings would imply that the mechanics of sperm Catsper channels may differ between testicular/epididymal spermatozoa and ejaculated spermatozoa, or even among species. Moreover, inhibition of *ABHD2* has been shown to suppress porcine sperm release from oviduct reservoir [63], which might associate the downregulation of *ABHD2* found in this study with the fact that sperm release from oviductal cells was not fully achieved, considering the timing of tissue sampling (24h post-insemination, absence of spontaneous ovulation, and lack of pre-ovulatory progesterone surge (P4 = 0.77 ± 0.35 pg/mL in peripheral blood) [30,48]. An increase in progesterone concentrations immediately prior to and following ovulation facilitates the release of spermatozoa from the oviduct epithelium so that they can reach the site of fertilization [63], a process that occurs sequentially [48], rather than all at once (as it is usually achieved in vitro [19]). The reason why many of the transcripts described in the present work are highly overrepresented in the mating group in comparison with the P1-AI group, despite both containing spermatozoa, may be explained by the fact that they represent two different procedures, natural mating and artificial insemination (AI). Not only does natural mating involve a series of behavioral, mechanical, and neuro-hormonal components which are not matched by AI, but mating also implies deposition of a larger number of spermatozoa compared to the AI of the sperm-peak fraction we used, which only represents about 25% of the total sperm ejaculated [64,65]. Conventional AI, where semen doses are customarily extended, implies a further reduction of sperm numbers to 2.5–3 billion spermatozoa but also of the amount of SP inseminated, which is approximately 30-times lower than that deposited during natural mating [64,65]. Such reductions, particularly of the amount of SP ought to be considered in the light of the present results. At first sight, sperm-free groups (SP-P1 and SP-Ejac) seemed to barely modify the molecular traits of the pig oviduct, as compared to the large numbers of DEGs elicited by sperm-containing treatments. However, we found some interesting results regarding the capacitation process. It is known that the SP contains ‘decapacitation factors’, mainly SP-proteins that coat and stabilize the sperm surface, likely to prevent premature capacitation, which are later removed to promote capacitation at a more appropriate time [66,67]. Seminal vesicles are believed to be responsible for the release of these decapacitating molecules that bind to the sperm membrane to inhibit capacitation. However, this phenomenon has been observed only in human and mice in vitro experiments that mixed capacitated spermatozoa with SP or candidate decapacitation factors [68,69,70]. In the pig, in vivo studies suggest that SP proteins are potentially involved in regulating sperm fertility [71,72].

Interestingly, we observed a down-regulation of *CATSPER1* and *CATSPER2* genes in oviduct cells when treated with sperm-free SP (SP-Ejac) in comparison with controls. Mutations of *CATSPER1* and *CATSPER2* genes have been related to male infertility in humans [73], and genetic deletion of any of the Catsper genes (*CATSPER* 1-4) resulted in loss of hyperactivated motility during the time of capacitation in mice [32,74,75]. Whether SP-molecules decapacitate spermatozoa through the inhibition of sperm Catsper signaling in the porcine oviduct has to the best of our knowledge, not been studied. In humans, although the mechanisms underlying these processes still remain to be fully elucidated, there is one molecule that appears to be involved in suppressing sperm motility and capacitation through the inhibition of Catsper channels: *CRISP1* [76], the first identified member of the highly evolutionarily conserved cysteine-rich secretory protein (CRISP) family. The role of *CRISP1* in the male tract has been extensively studied, where upon its binding to spermatozoa in the epididymis, it is carried with these cells into the female tract, acting as a decapacitating factor [77,78,79,80,81]. In mice, *CRISP1* inhibited membrane depolarization as well as Catsper signaling in epididymal spermatozoa, confirming the ability of the protein to block this sperm Ca^2+^ channel [82]. However, only scattered information is available regarding the presence of this protein in the mammalian female tract [83]. Ernesto et al. [82] was the first group to provide evidence that *CRISP1* is also expressed by oviduct cells and plays a role in fertilization by modulating hyperactivation, via modulation of key Catsper channels in human spermatozoa. The authors observed a reduction in sperm motility accompanied by an increase in *CRISP1*, which is in accordance with our findings, with *CRISP1* being upregulated in UTJ exposed to sperm-free seminal plasma (SP-P1) which suggests that *CRISP1* could be responsible for the inhibition of Catsper genes observed when oviduct cells were exposed to SP. The mechanism of action of *CRISP1* towards Catsper inhibition is not clear. It has been proposed that *CRISP1* has the ability to regulate protein tyrosine phosphorylation [78,79,84,85], thus giving one potential explanatory mode of action when blocking Catsper channels. However, further studies are required to clarify how an eventual interaction between *CRISP1 and CATSPER* functions. In addition, the *RNASE10* gene, associated in murine epididymal spermatozoa to loss in their ability to capacitate [86], appeared down-regulated in the pig UTJ exposed to sperm-free SP (both SP-P1 and SP-Ejac). It is tempting to consider that the pig pre-ovulatory oviduct responds to the entry of spermatozoa and/or SP with changes in the expression of genes that are related to the control of sperm capacitation. However, we must stress that these responses are not directional nor simple, and that the treatments used include confounding factors that need to be considered. To start with, although this in-vivo experiment seems to demonstrate the influence of SP in regulating the capacitation process through molecular signaling in the oviduct, all treatments had SP, albeit in varying amounts. The major differences were: (i) the presence or absence of spermatozoa as well as (ii) the relative amount of SP-proteins present, considering the P1-fraction is the one containing the lowest amount of major SP proteins of the ejaculate [8]. Thus the presence of spermatozoa implies an up-regulation of *AKAPs*, *CATSPERs,* and *CABYR* genes, thus positively regulating the Catsper channel, while the SP downregulated *PKIs* and *CRISP1* genes, which seem inhibitors of the Catsper channels is not an easy equation to handle. Although we have studied tubal samples, dissected to primarily sample the mucosa, it is impossible to fully ensure that all of the samples contained no spermatozoa. Whether sperm presence could per-se influence the gene expression is hard to establish, considering their relative transcriptomic inactivity.

Interestingly, in samples of which were incidentally also examined as comparison in this study (data not shown), the microarray platform revealed the alteration of many transcripts among semen-treatments in an upper segment of the oviduct (ampulla), despite the immense majority of spermatozoa are known to be located in the UTJ-isthmic reservoir during the time of sample collection (24 h after inseminations) [87]. Molecular signaling have been already studied in the site of fertilization, but at a later stage, post-ovulation, when both male and female gametes are transported to [88]. Some of the genes found upregulated in the ampulla at a post-ovulation stage [88] were found downregulated in the pre-ovulatory ampulla after mating (e.g., *SPP1*), while others appeared to be upregulated in in ampulla in both pre-and post-ovulatory periods (e.g., *ITIH4* and *TSHZ2*). Additionally, in the ampullar samples we found an overall downregulation of fertilizing-related genes after mating and P1-AI, such as *DNAJA1, DLD,* and *TEKT2*, described before for their role in sperm maturation, motility and capacitation [89,90,91], or *CCT2, CCT4, CCT7* (mating and AI)*, IZUMO1, ZP4,* and *ZP2* (only mating), implicated in gamete recognition and sperm-binding to the ZP [92,93,94,95], suggesting that the oviduct itself is capable of suppressing the signals that enables spermatozoa to fertilize the oocyte, ensuring an adequate and efficient transition of these processes once the mature oocyte has reached the ampulla. Whether such findings are transient during pre-ovulation to change after ovulation has occurred, remains to be studied.

Regarding the presence of differential amounts of SP in all groups, the differences consisted in all SP-proteins being present in mating and its corresponding Ejac-SP, while the P1-AI and its SP-counterpart represented the SP fraction with the lowest contribution from the seminal vesicles, thus having the lowest amount of major SP proteins (spermadhesins) but the highest amounts of cauda epididymal fluid, which is considered the most effective milieu to maintain sperm quiescence, besides electrolyte composition [96]. In this regard, we could hypothesize that this scenario of highly abundant anti-capacitating factors masking pro-capacitation signals may be related to the fact that 24 h post insemination, most spermatozoa are maintained in a non-capacitated stage within the oviductal reservoir awaiting until ovulation takes place. It is concluded that the oviduct promotes, via a differential regulation of Catsper channels, pro- and anti-capacitation molecular responses. Whether the action of the genes particularly stimulated in the sperm reservoir/adjacent isthmus by the SP is to prevent premature massive capacitation prior to ovulation ought to be further studied.

## 4. Materials and Methods

### 4.1. Experimental Design

The transcriptomics of the pre/peri-ovulatory pig oviduct reservoir (UTJ and isthmus), was studied in a total of 20 pre-ovulatory sows, 24 h after the deposition of semen (i.e., spermatozoa and SP) or of sperm-free SP. Multiparous sows displaying standing oestrus in the presence of a boar were equally and at-random allotted to one of three groups, namely:

**Control** (*n* = 4), where females were cervically infused with 50 mL of the protein-free extender “Beltsville Thawing Solution” (BTS) [97],

**Exposure to semen** (spermatozoa and the accompanying SP) (*n* = 8), sub-divided as natural mating (M, *n* = 4), with sows each mated with a single male; or artificial cervical deposition (AI) of the sperm-peak ejaculate fraction e.g., the first 10 mL of the sperm-rich fraction (P1, extended with BTS to 50 mL) (P1-AI, *n* = 4),

**Exposure to sperm-free SP** (*n* = 8), sub-divided as the SP of the entire ejaculate (SP-Ejac, 50 mL, *n* = 4) or only the SP from the P1 fraction (SP-P1, pool, 50 mL, *n* = 4), either via cervical AI.

Samples from 2 different segments of the oviduct: the utero-tubal junction (UTJ) and isthmus (Isth) were surgically retrieved under general anesthesia, 24 h after the mating/AIs [10].

### 4.2. Animal Management Including Ethics Statement

Pigs of Swedish Landrace breed were recruited from a controlled breeding farm, as weaned sows (parity 1–3, *n* = 20) and young mature boars (9–11 months, *n* = 5) of good semen quality (< 100 mL volume, >60 × 10^9^ total sperm number, >70% sperm motility, and >75% morphologically normal-looking spermatozoa, controlled weekly) [10]. Throughout all experiments, animals were handled carefully to avoid any unnecessary stress. The animals were individually kept in separate pens at the Translational Medicine Centre (TMC/CBR-3) of Linköping University under controlled temperature and light regimes (12 h:12 h light/dark cycle). Pigs were fed with commercial feedstuff (Lantmännen, Stockholm, Sweden) according to national standards provided with water ad libitum and receiving the same management.

All animal husbandry and experimental handling was performed in compliance with the European Community (Directive 2010/63/EU) and current Swedish legislation (SJVFS 2017:40), being approved in advance by the “Regional Committee for Ethical Approval of Animal Experiments” (Linköpings Djurförsöksetiska nämnd) in Linköping, Sweden (permits no. 75-12 and no. ID1400).

### 4.3. Semen Collection and SP Harvesting

Ejaculates and the specific P1-fraction, collected using the gloved-hand method, with at least 70% motile and 75% morphologically normal-looking spermatozoa immediately after collection, were used. The SP was harvested either from the whole ejaculate or from the sperm-peak P1-fraction after double centrifugation at 1500× *g* for 10 min, and microscopically checked for presence of spermatozoa. The harvested sperm-free crude SP was stored at −20 °C until used.

### 4.4. Handling of Sows

Detection of estrus of the sows was performed twice daily, beginning one day after weaning, during their snout-to-snout contact with adjacent located mature boars, while testing for standing estrus reflex by applying back-pressure. When sows showed standing estrus reflex they were considered to be on the first day of behavioral estrus and then mated (M) or cervical AI with disposable commercial AI-catheters (Minitüb, Munich, Germany) (control, P1-AI, SP-P1, and PS-Ejac).

### 4.5. Collection of Oviductal Samples

All sows were subjected to mid-ventral laparotomies to collect the tissue samples 24 h after mating/inseminations (pre-/peri-ovulation period), as previously described [10]. Briefly, sows were sedated by the i.m. administration of a mixture of 5 mg dexmeditomedine (Dexdomitor, Orion Pharma Animal Health, Sollentuna, Sweden) and 100 mg tiletamine hydrochloride/zolazepam hydrochloride (Zoletil vet, Virbac A/S, Kolding, Denmark) followed by anesthesia induced i.v. with sodium thiopental (Abbot Scandinavia AB, Solna, Sweden, 7 mg/kg bw), and maintained with isoflurane (3.5–5%, Baxter Medical AB, Kista, Sweden) administered via a tracheal cuffed tube by a close-circuit PVC-ventilator (Servo ventilator 900 D, SIEMENS-ELEMA AB, Solna, Sweden). The number of pre-ovulatory follicles per sow was 22.3 ± 7.3 (mean ± SD), without significant differences between sow-groups. Peripheral blood plasma was analyzed (ELISA) for progesterone (P_4_) and estradiol 17ß (E_2_) contents, confirming the sows were in pre-/peri-ovulatory estrus (P_4_ = 0.77 ± 0.35 pg/mL; E_2_ ranging 294.2–376.50 ± 27.76 pg/mL, *p* > 0.05 among sows/groups). Oviductal segments (UTJ and Isth) were immediately retrieved, opened to expose the mucosa and stored at –80°C in RNAlater (Ambion, Thermo Fisher Scientific Baltics UAB, Vilnius, Lithuania) until analyzed.

### 4.6. Transcriptome Analysis

Total RNA was isolated from tissue samples using Trizol reagent (Invitrogen, Carlsbad, CA, USA) and quality assessment was performed using an Agilent 2100 Bioanalyzer (Agilent Technologies, Santa Clara, CA, USA) according to the manufacturer’s instructions. The RNA integrity number (RIN) values obtained were in the range of 8 to 10, which guarantied the homogeneity and high quality of the samples. Equal amounts of total RNA (250 ng) from each sample were used to make cDNA using GeneChip^®^ Whole Transcript Plus reagent kit (Affymetrix, Santa Clara, CA, USA) following the manufacturer protocol. cDNA was then hybridized and loaded on the array chip (GeneChip^®^ Porcine Gene 1.0 ST Array, Affymetrix Inc., 3420 Central Expressway, Santa Clara, CA 95051, USA), incubated at 45 °C under 60 rotations per min, for 16 h. The hybridized cartridge array chip was then unloaded and subjected to washing and staining using a GeneChip^®^ Fluidics Station 450 (Affymetrix), to be finally scanned using the Affymetrix GeneChip^®^ scanner GCS3000 [10].

### 4.7. Analysis of Microarray Data and Bioinformatics

The intensity data of each array chip was processed using the robust multi-array average (RMA) normalization, computing average intensity values by background adjustment, quantile normalization among arrays and finally log_2_ transformation to extract the expression values of each transcript in the probe set, as implemented in the official Transcriptome Analysis Console (TAC; version 4.0) from Affymetrix. The statistical analysis of the normalized gene expression data was performed using a linear model using the empirical Bayes’ approach as implemented in the package “limma” was used to calculate differentially expressed transcripts using a Benjamini-Hochberg (q < 0.05) correction to control for multiple testing to control type-I errors [98] and a fold change (FC) >1 or <−1.

### 4.8. Enrichment Analysis

The data was then analyzed using the Database for Annotation, Visualization and Integrated Discovery (DAVID 6.7). The differentially expressed genes (*p* < 0.05) were screened for additional molecular functions with the protein knowledge base of the UniProt Consortium [99]. Graphical illustration of overrepresented GO terms was produced with the Cytoscape v3.0.0 application CluePedia v2.0.3 [100]. Schematic representation of Catsper channels regulation was performed with Biorender^®^ and SmarArt^®^ softwares.

## 5. Conclusions

Altogether, the present findings point out that the presence of spermatozoa or of relative amounts of SP in the pre-ovulatory pig oviduct promotes pro- and anti-capacitation molecular responses via a differential regulation of Catsper channels. It is postulated that genes particularly stimulated in the sperm reservoir/adjacent isthmus by the SP would be to prevent premature massive capacitation prior to ovulation.

## Figures and Tables

**Table 1:** Subset of altered transcripts potentially involved in sperm capacitation and/or fertilization in UTJ and Isth segments of the oviduct 24 h after mating or P1-AI.

***MATING***							
***UTJ***				**ISTH**			
**Gene ID**	**Fold Change**	***p*-value**	**Description**	**Gene ID**	**Fold Change**	***p*-value**	**Description**
ABHD2	−1.66	0.004	Monoacylglycerol lipase protease	*ABHD2*	−2.6	0.0003	Monoacylglycerol lipase protease
CATSPER2	1.53	0.01	Cation channelsperm-associated protein 2	*CATSPERγ*	1.3	0.03	Cation channelsperm-associated subunit gamma
CATSPERγ	1.56	0.006	Cation channelsperm-associated subunit gamma	*ING2*	1.25	0.003	Inhibitor of growth protein 2
GAS8	1.37	0.002	Dynein regulatory complex subunit 8	*AAAS*	−1.38	0.03	Aladin WD repeat nucleoporin
RLN2	−1.16	0.02	Prorelaxin precursor	*B4GALT1*	−1.89	0.0005	Beta-1,4-galactosyltransferase
TEKT2	1.62	0.03	Tektin-2 non-motor microtubule binding protein	***BCL2L1***	1.28	0.009	Anti-apoptotic signaling molecule
TEKT3	1.45	0.04	Tektin-3 non-motor microtubule binding protein	*CCT3*	−1.19	0.04	T-complex protein 1 subunit gamma
B4GALT1	−1.69	0.0009	Beta-1,4-galactosyltransferase	*EHMT2*	1.18	0.02	Histone-Lysine N-methyltransferase
BCL2L1	1.37	0.007	Anti-apoptotic signaling molecule	***MFGE8***	−4.37	<0.0001	Lactadherin membrane-bound signaling molecule
MFGE8	−2.23	0.004	Lactadherin membrane-bound signaling molecule	*NR2F2*	1.73	0.001	C4 Zinc finger nuclear binding receptor
RNASE10	−1.15	0.03	Inactive ribonuclease-like protein 10	*SPINK2*	1.33	0.03	Serine protease inhibitor Kazal type 2
SPAM1	−1.21	0.04	Sperm adhesión member	*TARBP2*	−1.26	0.004	RISC-loading complex subunit 2
SPEF2	1.61	0.03	Sperm flagelar protein 2	***ZP4***	−4.09	<0.0001	Zona pellucida sperm-binding protein 4
TARBP2	−1.47	0.001	RISC-loading complex subunit 2	*AKAP11*	1.18	0.04	A-Kinase Anchor protein 11
TRPC3	1.71	0.003	Short transitient receptor potential channel 3	*AKAP12*	1.55	0.02	A-Kinase Anchor protein 12
ZP4	−1.76	0.03	Zona pellucida sperm-binding protein 4	***PKIA***	−3.4	0.0002	cAMP-dependent kinase inhibitor alpha
AKAP11	1.43	0.007	A-Kinase Anchor protein 11	*CABYR*	1.22	0.01	Calcium-binding tyrosine phosphorylation-regulated protein
AKAP13	1.37	0.007	A-Kinase Anchor protein 13				
PKIA	−2.33	0.02	cAMP-dependent kinase inhibitor alpha				
CABYR	1.33	0.007	Calcium-binding tyrosine phosphorylation-regulated protein				
**P1-AI**							
***UTJ***				**ISTH**			
CACNA1I	1.56	0.006	Voltage-dependent L-type calcium channel	ACRBP	1.38	0.04	Acrosin-binding protein
CFTR	−2.38	0.009	ATP-binding cassette transporter	CATSPERγ	1.42	0.008	Cation channelsperm-associated subunit gamma
DPCD	−1.49	0.04	Deleted in primary ciliary dyskinesia	ING2	1.17	0.03	Inhibitor of growth protein 2
SPAG6	−2.58	0.03	Sperm-associated antigen 6	AQN-1	−1.336	0.04	Carbohydrate-binding protein
HOXD9	1.52	0.02	Homeobox protein 9	PRDM14	1.19	0.04	PR domain Zinc finger protein 14
HOXD10	2.42	0.04	Homeobox protein 10	TARBP2	−1.18	0.01	RISC-loading complex subunit 2
SPA17	−1.7	0.03	Sperm Surface protein	ZP4	−3.01	0.0007	Zona pellucida sperm-binding protein
TARBP2	−1.25	0.02	RISC-loading complex subunit 2				
TRPC3	1.65	0.01	Short transient receptor potential cannel 3				
AKAP11	1.57	0.009	A-Kinase Anchor protein 11				
AKAP13	1.4	0.04	A-Kinase Anchor protein 13				
PKIB	−1.81	0.01	cAMP-dependent kinase inhibitor beta				

Transcripts showing an FDR < 0.05 are marked by bold font.

**Table 2:** Subset of altered transcripts potentially involved in sperm capacitation and/or fertilization in UTJ and Isth segments of the oviduct 24 h after sperm-free seminal plasma (SP) exposal (SP-P1 or SP-Ejac).

***SP-P1***							
***UTJ***				**ISTH**			
**Gene ID**	**Fold Change**	***p*-value**	**Description**	**Gene ID**	**Fold Change**	***p*-value**	**Description**
INSL6	1.39	0.0007	Insulin-like 6 peptide	CACNA1D	1.65	0.0008	Voltage-dependent L-type calcium channel
RNASE10	−1.21	0.02	Inactive ribonuclease-like protein 10	TEKT3	−1.14	0.04	Tektin-3 non-motor microtubule binding protein
SLC26A8	1.28	0.02	Anion transporter 1	EHHMT2	1.16	0.04	Histone-Lysine N-methyltransferase
CRISP1	1.3	0.04	Custein-rich secretory protein 1	HOXA11	−1.31	0.02	Homeobox protein A-11
PLCZ1	1.17	0.02	Calcium-binding protein phospholipase signaling molecule	SYCP2	1.92	0.01	Synaptonemal complexprotein 2
TRPC3	1.45	0.04	Short transient receptor potential cannel 3	TARBP2	−1.33	0.0007	RISC-loading complex subunit 2
***SP-Ejac***							
***UTJ***				**ISTH**			
CATSPER1	−1.37	0.01	Cation channelsperm-associated protein 1	AAAS	1.16	0.04	Aladin WD repeat nucleoporin
RLN2	−1.22	0.04	Prorelaxin precursor				
RNASE10	−1.16	0.03	Inactive ribonuclease-like protein 10				
